# Habitat Affects the Chemical Profile, Allelopathy, and Antioxidant Properties of Essential Oils and Phenolic Enriched Extracts of the Invasive Plant *Heliotropium Curassavicum*

**DOI:** 10.3390/plants8110482

**Published:** 2019-11-07

**Authors:** Ahmed M. Abd-ElGawad, Abdelsamed I. Elshamy, Saud L. Al-Rowaily, Yasser A. El-Amier

**Affiliations:** 1 Plant Production Department, College of Food & Agriculture Sciences, King Saud University, P.O. Box 2460, Riyadh 11451, Saudi Arabia; srowaily@ksu.edu.sa; 2 Department of Botany, Faculty of Science, Mansoura University, Mansoura 35516, Egypt; yasran@mans.edu.eg; 3 Chemistry of Natural Compounds Department, National Research Centre, 33 El Bohouth St., Dokki, Giza 12622, Egypt; elshamynrc@yahoo.com; 4 Faculty of Pharmaceutical Sciences, Tokushima Bunri University, Yamashiro-cho, Tokushima 770-8514, Japan

**Keywords:** essential oil, bio-herbicides, phytotoxicity, invasive plants, weed control, hexahydrofarnesyl acetone

## Abstract

The variation in habitat has a direct effect on the plants and as a consequence, changes their content of the bioactive constituents and biological activities. The present study aimed to explore the variation in the essential oils (EOs) and phenolics of *Heliotropium curassavicum* collected from the coastal and inland habitats. Additionally, we determined their antioxidant and allelopathic activity against the weed, *Chenopodium murale*. Fifty-six compounds were identified as overall from EOs, from which 25 components were identified from the coastal sample, and 52 from the inland one. Sesquiterpenes were the main class in both samples (81.67% and 79.28%), while mono (3.99% and 7.21%) and diterpenes (2.9% and 1.77%) represented minors, respectively. Hexahydrofarnesyl acetone, (-)-caryophyllene oxide, farnesyl acetone, humulene oxide, farnesyl acetone C, and nerolidol epoxy acetate were identified as major compounds. The HPLC analysis of MeOH extracts of the two samples showed that chlorogenic acid, rutin, and propyl gallate are major compounds in the coastal sample, while vanilin, quercetin, and 4′,7-dihydroxyisoflavone are majors in the inland one. The EOs showed considerable phytotoxicity against *C. murale* with IC_50_ value of 2.66, 0.59, and 0.70 mg mL^−1^ for germination, root, and shoot growth, respectively from the inland sample. While the coastal sample attained the IC_50_ values of 1.58, 0.45, and 0.66 mg mL^−1^. MeOH extracts revealed stronger antioxidant activity compared to the EOs. Based on IC_50_ values, the ascorbic acid revealed 3-fold of the antioxidant compared to the EO of the coastal sample and 4-fold regarding the inland sample. However, the ascorbic acid showed 3-fold of the antioxidant activity of the MeOH extracts of coastal and inland samples. Although *H. curassavicum* is considered as a noxious, invasive plant, the present study revealed that EO and MeOH extracts of the *H. curassavicum* could be considered as promising, eco-friendly, natural resources for antioxidants as well as weed control, particularly against the weed, *C. murale*.

## 1. Introduction

From ancient times, most of the world’s population use traditional medicinal plants as the first target of medicines because of the revolutions in traditional philosophy [1]. Around 40%–50% of used pharmaceutical drugs around the world are derived from natural resources due to several side effects of synthetic chemical drugs in most cases [2]. From the start of civilization and due to the bioactivities, many traditional usages of the essential oils (EOs) have been used overall the world [3]. The EOs are very important as they integrated into several industries such as pharmaceutical, cosmetic, and nutritional products [4]. Furthermore, the EOs were characterized to have several biological activities including antimicrobial, antioxidant, anti-cancer, anti-inflammatory, insecticidal, and allelopathic [4,5,6,7,8]. Additionally, the EOs were characterized to control the biodeterioration of archaeological sites due to their activity against degradable microorganisms [9,10,11].

The EOs are a mixture of volatile molecules that include different classes of compounds with concentration variability and characterized by the preponderance of terpenes especially, mono and sesquiterpenes [3,12]. On the other hand, EOs play important roles in the ecosystem where they act as signals in plant communication [13], protect the plant from herbivores [14], and attract insects for pollination [15]. 

Invasive plants are reported as one of the most serious problems in the ecosystem worldwide [16]. One of the mechanisms of plant invasion is the integration of plant bioactive chemicals (allelochemicals) into chemical weapons of invasion [17]. Allelochemicals are bioactive secondary metabolites embracing various groups, for example, phenolic acids, glucosinolates, flavonoids, essential oils [18].

*Heliotropium curassavicum* (salt heliotrope) is an annual plant of family *Boraginaceae*. It is native to America, Argentina, Indies, and Hawaii, but it is one of the invasive plants in Africa, Europe, and Asia [19]. In Egypt, this pant is considered an invasive plant in the northern coastal belt of the Mediterranean Sea [20], while recently it was observed to colonize disturbed habitats inside the Nile Delta, Egypt (personal observation). This plant has the ability to shift its reproduction from vegetative to sexual and vice versa, which enables it to colonize multiple habitats [21]. The variation of the environmental conditions usually leads to changes in the bioactive ingredients of the plants [3,22], where it is considered as an adaptive mechanism as well as these bioactive compounds enable the plants to invade and colonize new habitats [17].

Several compounds were isolated and identified from *Heliotropium* species such as pyrrolizidine alkaloids [23,24], flavonoids [25,26], triterpenes [27], and benzofurans [28]. Numerous biological activities of extracts and isolated compounds from *Heliotropium* plants were documented like antimicrobial [27], wound healing [24], antioxidant [23,25,28], antineoplastic, cytotoxic activities [26], and anti-inflammatory activity [29]. However, the bioactive compounds of *H. curassavicum* is not well studied, and to our knowledge, no study explores the chemical composition or the bioactivities of its EO.

The hypotheses of the present work were (a) does the ability of *H. curassavicum* to colonize different habitats lead to changes in their bioactive constituents? (b) Are the changes in the bioactive composition with habitat, coupled with changes in their bioactivities? Therefore, the present study aimed to (i) characterize the chemical compound of the EOs from *Heliotropium curassavicum* collected from two heterogeneous habitats, (ii) investigate the HPLC-quantitative and qualitative phenolic profile of the MeOH extracts of the two samples, (iii) determine the antioxidant activity of the EOs and the MeOH extracts, and (iv) evaluate the allelopathic activity of the EOs and the MeOH extracts against the nuisance weed, *Chenopodium murale*.

## 2. Results and Discussion

### 2.1. Soil Physico-Chemical Parameters

The soil analysis revealed significant variations between the coastal and inland habitat (Table 1). Soil texture showed a significant difference between the coastal and inland habitat (*p* = 0.008), where coastal soil is sandy, while the inland soil has a high content of clay. Moreover, the coastal soil attained high content of salt (electrical conductivity, EC = 1.68 mS cm^−1^) compared to the inland soil (EC = 0.57 mS cm^−1^). In addition, a significant difference was observed for organic carbon content, total dissolved phosphorus, and total nitrogen (Table 1).

### 2.2. Chemical Composition of the EOs 

Two samples of *H. curassavicum* from two different habitats, coastal and inland, were subjected to hydro-distillation using the Clevenger-type apparatus for 3 h and afforded two yellow oils samples with volumes of 0.026 and 0.021 mL, respectively. This variation in the quantity of the EOs between the coastal and inland samples could be ascribed to the effect of the habitat, particularly the salinity effect (Table 1). It was described that the treatment of thyme plants with 100 mM NaCl induced the production of the EO by 16.11% [30].

**Figure 1:**
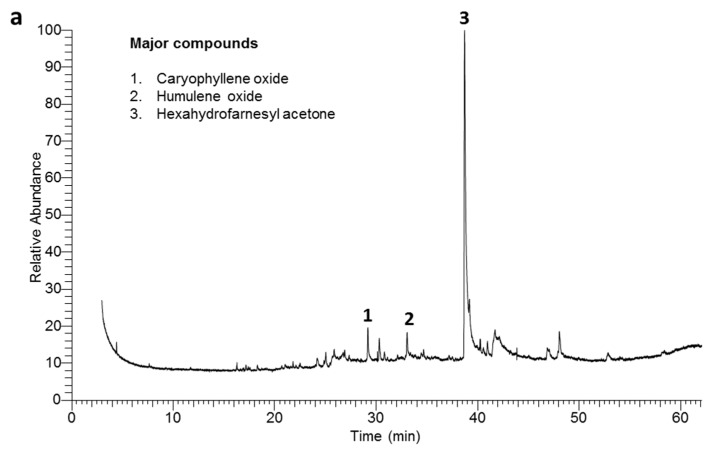

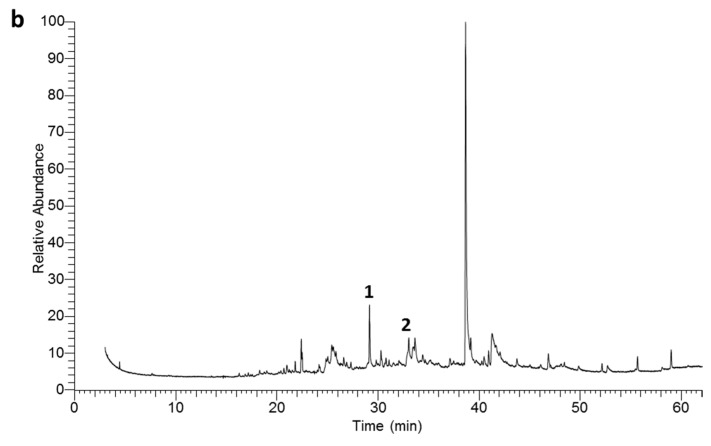
GC-MS chromatogram of the essential oil derived from the (**a**) coastal habitat sample of *H. curassavicum* and (**b**) from the inland habitat sample. The peaks of major compounds are numbered (1–9).

The two EO samples were analyzed via GC/MS. The chromatograms exhibiting the main compounds of overall identified compounds in coastal and inland samples are presented in Figure 1. Moreover, all the identified compounds, representing 100% of the total mass of the EOs, are listed in detail, as shown in Table 2. Fifty-six compounds were characterized overall from the two samples, including 25 compounds from coastal while 52 from inland were identified. The results of the chemical characterization of the EOs of the two samples showed that the two EOs comprise monoterpenes, sesquiterpenes, diterpenes, hydrocarbons, in addition to other compounds. 

The EOs of the two samples, coastal and inland, were characterized by the preponderance of the sesquiterpenes (81.67% and 79.28%, respectively). These compounds were identified and categorized as sesquiterpene hydrocarbons (10.31% and 12.67%) and oxygenated sesquiterpenes (71.36% and 66.51%). From the above result, the oxygenated sesquiterpenes represented the main ingredients of the two samples but the coastal sample was higher than the inland one. At the same time, the sesquiterpene hydrocarbons in the inland sample were higher than the coastal one. This small difference between the two samples in all constituents, especially sesquiterpenes as a major class, may exhibit the effects of environmental factors of each plant sample. Similar observations were reported in previous studies [3,31].

From the all identified sesquiterpenoids, hexahydrofarnesyl acetone (50.39% and 35.82%), (-)-caryophyllene oxide (6.41% and 7.86%), humulene oxide, farnesyl acetone (0% and 7.17%), (5.01% and 4.02%), farnesyl acetone C (5.21% and 0.99%), and nerolidol epoxy acetate (3.0% and 1.64%) represented the major compounds. Hexahydrofarnesyl acetone was the main component of EOs of the two samples but it is observed that its concentration in the EO of coastal sample is higher than the inland one by around 15%. This result is in complete agreement with previously reported EO of Nigerian plant, *Heliotropium indicum* [32]. Additionally, farnesyl acetone C represented a major compound in the coastal sample, while it is a minor in the inland one. In oppositely, farnesyl acetone was a major compound in the inland sample but completely disappeared in the coastal one.

Monoterpenes represented a considerable class of identified compounds in the two samples, but the inland one (7.21%) is richer than the coastal (3.99%). The oxygenated monoterpenes represented the major identified compound with a concentration of 6.49% in the inland sample compared to 3.2% for the coastal one. The two oxygenated monoterpenes, *trans*-*α*-ionone (2.05%), and *cis*-*α*-ionone (2.21%) were identified as major compounds in the inland sample only while *trans*-p-menthan-7-ol (2.34% and 1.81%) was characterized in both samples. Only two monoterpene hydrocarbons, *α*-pinene (0.4%), and *β*-elemene (0.32%) represented the overall compounds identified in the inland sample, while *α*-pinene (0.79%) is the only one in the coastal one. 

Diterpenes were rarely documented as major compounds in EOs of most plants [33]. Two diterpenoids including, one diterpene hydrocarbon, phytan (1.07% and 0.72%), and one oxygenated diterpene, phytol (1.83%, and 1.05%), were characterized as overall diterpenes (2.9% and 1.77%) in EOs of coastal and inland samples of *H. curassavicum*. Phytol was already reported as a major volatile diterpene from EOs derived from some *Heliotropium* plants such as Nigerian and Thailand *H. indicum* [32,34], *H. europaeum* [35].

The hydrocarbons represented the second major class of compounds in coastal and inland plant samples with concentrations of 10.78% and 10.97%, respectively. The majority of the hydrocarbons were documented in the EOs of several plants around the world and herein from some *Heliotropium* species such as *H. indicum* [32,34], *H. europaeum* [35]. Non-oxygenated hydrocarbons were characterized as major compounds of overall hydrocarbons with concentrations of 9.71% and 8.35% in addition to minor oxygenated hydrocarbons with concentrations of 1.07% and 2.62%. 

Finally, one benzopyrane compound namely, dihydroedulan II, was identified in EOs of the two plant samples, coastal (0.66%) and inland (0.32%). While the phenolic compound, 2-tetra-butyl-4-isopropyl-5-methylphenol, with a concentration of 0.55% was characterized in EO of the inland sample of *H. curassavicum*. All these mentioned data exhibited that the inland sample of *H. curassavicum* is more diverse in the EO components than the coastal one, where this observation could be attributed to the effect of habitat. *H. curassavicum* is reported to bloom well in salty soils in salt marshes, sandy beaches, and disturbed coastal sites [19]. Therefore, when this plant invades new areas, its chemical profile changes as a way of adaptation to the new environmental conditions or as a chemical weapon for competition and invasion [17,36].

### 2.3. Phenolic Profile of 70% MeOH Extract 

**Figure 2:**
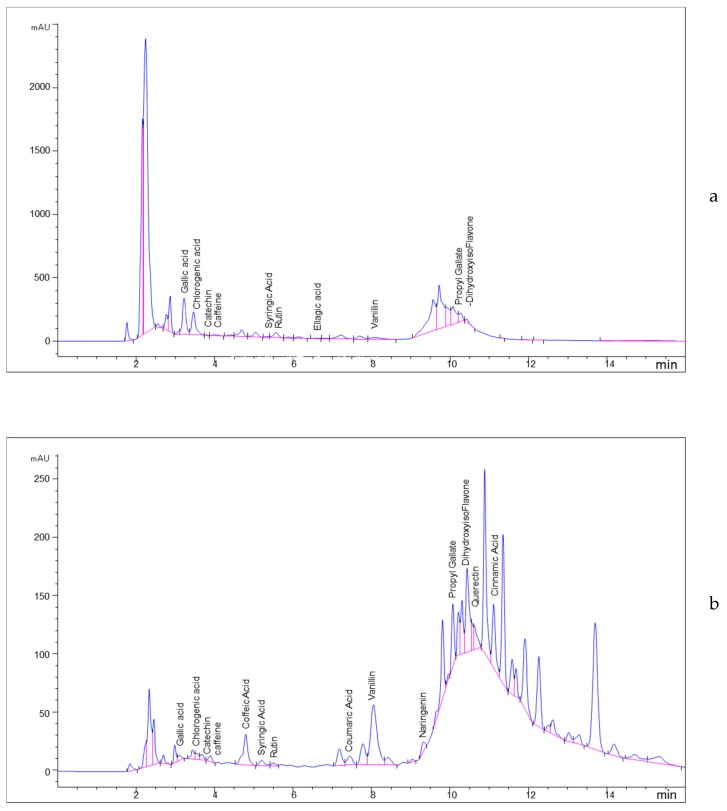
HPLC chromatogram of the MeOH extract from (**a**) the coastal habitat sample of *H. curassavicum* and (**b**) from the inland habitat sample.

The *Heliotropium* species were characterized by the flavonoids [25,26]. An external authentic mixture was chromatographed according to the HPLC conditions described in the experimental section. At the same condition, a concentration of 1 mg/1 mL from the 70% MeOH extract was subjected to HPLC [37]. The chromatograms of the HPLC analysis of the MeOH extract from the coastal and inland samples were recorded at 278 nm and presented in Figure 2. All the identified compounds from the two *H. curassavicum* samples, coastal and inland, are listed in Table 3. Ten polyphenolic compounds were identified from the coastal plant sample while 14 compounds were characterized from the inland one.

From MeOH extract of the coastal sample, four phenolic acids, gallic acid, chlorogenic acid, syringic acid, and ellagic acid, alongside three phenolic acid derivatives, caffeine, vanillin, and propyl gallate, were characterized. Chlorogenic acid was identified as a major phenolic acid with a concentration of 1198.24 µg g^−1^ while syringic acid represented a minor one with a concentration of 16.97 µg g^−1^.

By the same method, six phenolic acids including, gallic acid, chlorogenic acid, caffeic acid, syringic acid, ellagic acid, and coumaric acid, in addition to three derivatives of phenolic acid including, caffeine, vanillin, and propyl gallate, were characterized from the inland plant sample. From all identified phenolic acids and their derivatives, vanillin and caffeic acid were characterized as major compounds in this sample with concentrations of 1284.61 and 452.87 µg g^−1^, respectively. 

Three flavonoids, catechin, rutin, and 4′,7-dihydroxyisoflavone, were identified from the coastal plant sample with a majority of rutin with a concentration of 389.65 µg g^−1^. While, in the inland sample, five flavonoids, catechin, rutin, naringenin, quercetin, and 4′,7-dihydroxyisoflavone, were identified. On the contrary, 4′,7-dihydroxyisoflavone and quercetin represented the main flavonoids in the inland sample with concentrations of 721.52 and 603.63 µg g^−1^, respectively.

The results exhibited that the inland sample of *H. curassavicum* is richer than the coastal one in polyphenolic constituents. Naringenin with a high concentration in the inland sample was in complete agreement with the fact of high distribution of this compound in different species of this genus such as *H. subulatum* [25], *H. sclerocarpum* [28], and *H. taltalense* [28]. For the phenolic acids and their derivatives, some reported stated the isolation of some derivatives such as filifolinol, filifolinylsenecionate, filifolinone, and filifolinoic acid, etc. [38,39]. All these reports described that the plants belonging to this genus are very rich with phenolic acids and their derivatives. Herein, the HPLC phenolic characterization of *H. curassavicum* was totally coordinated with this fact by the high concentration of phenolic compounds, especially chlorogenic acid, vanillin, caffeic acid, and propyl gallate. 

The results exhibited that the effects of the habitat of the plant is clear in the differences in quantity and quantity of phenolic compounds. It was reported that the total phenolic content of *Thymus vulgaris* and *Thymus daenensis* was induced by 20% after the application of 60 mM NaCl and in consequence improved the antioxidant capacity [40]. It was reported that salinity induces the production of phenolic compounds as a mechanism of acclimatization to stressful conditions [41].

### 2.4. Allelopathic Activity

#### 2.4.1. Allelopathic Effect of the EOs

**Figure 3:**
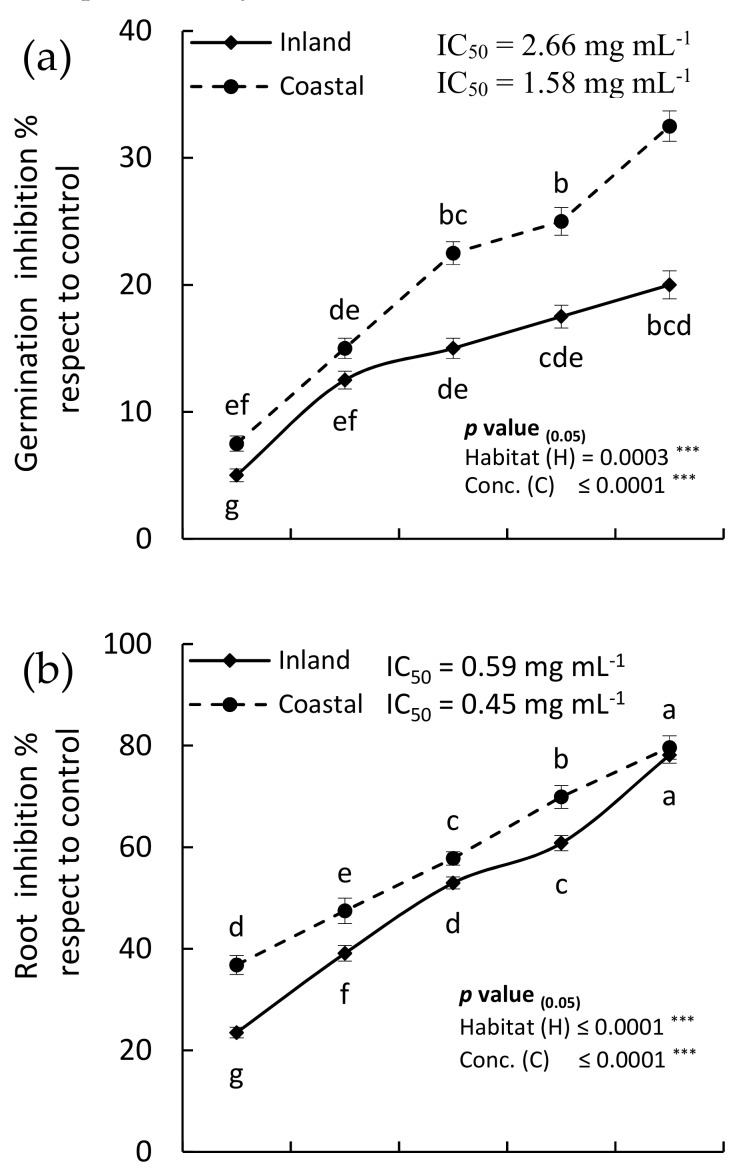

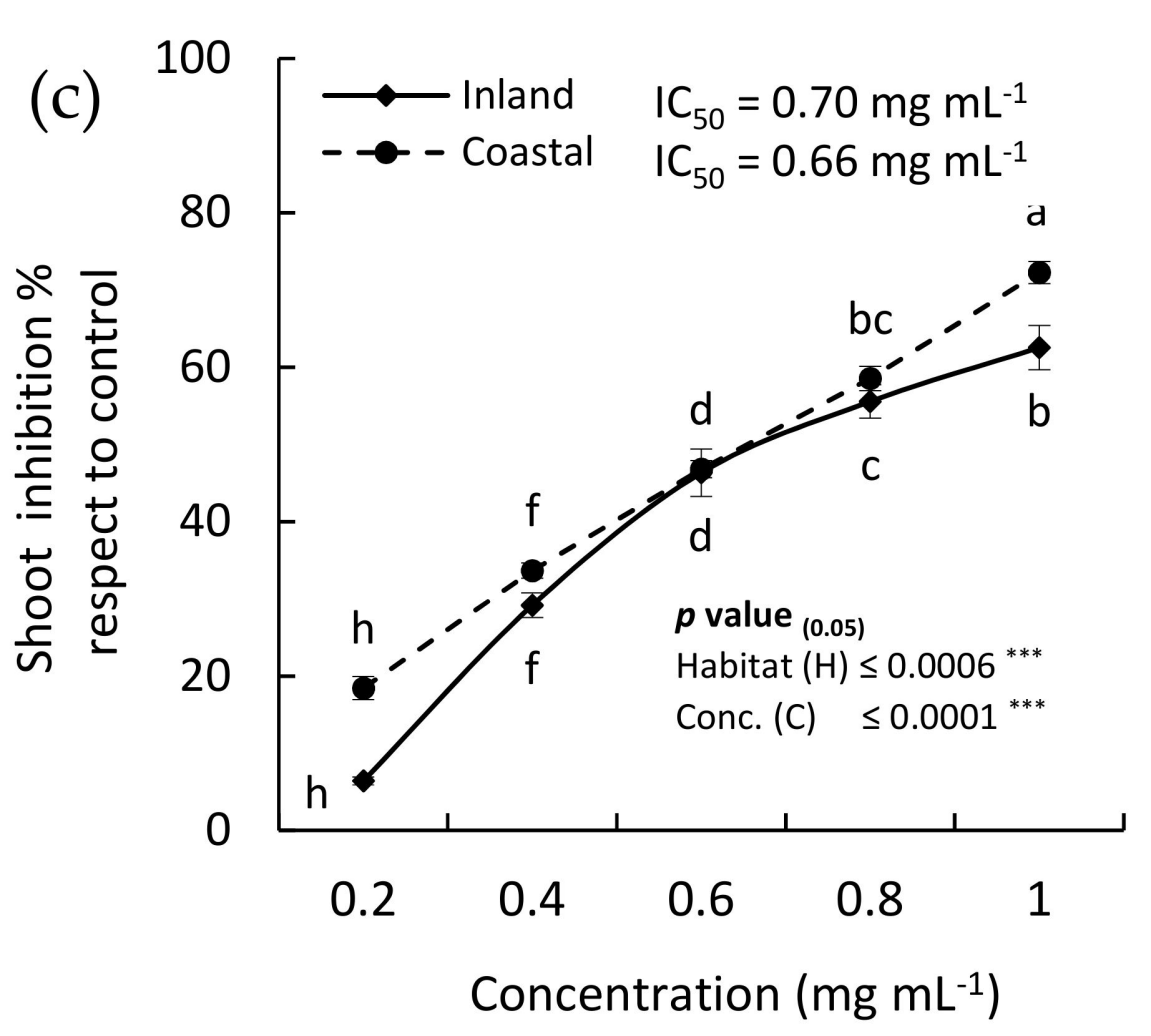
Allelopathic inhibitory effect of various concentrations of essential oil extracts of the aerial parts of *Heliotropium curassavicum* collected from coastal and inland habitats on (**a**) the germination, (**b**) root, and (**c**) shoot growth of *Chenopodium murale*. Different letters within each measurement mean values of significant variation at *p* ≤ 0.05. IC_50_ is the concentration extract required for 50% inhibition.

The EOs of the two samples (coastal and inland) from *H. curassavicum* aerial parts showed a significant allelopathic inhibitory effect on the germination and seedling growth of the weed *C. murale* (Figure 3). The inhibitions of both germination and seedling growth were significantly dose dependent. At the highest concentration (1 mg mL^−1^), the germination was inhibited by 20.00% and 32.5% for inland and coastal samples, respectively. Moreover, the root growth of the seedling was reduced by 78.11% and 79.62%, while the shoot was reduced by 62.55% and 72.26%, for inland and coastal samples, respectively. The IC_50_ values of the EO from the inland sample was 2.66, 0.59, and 0.70 mg mL^−1^ for germination, root, and shoot growth, respectively, while the coastal samples attained the IC_50_ values of 1.58, 0.45, and 0.66 mg mL^−1^. It is clear that root was affected more than the shoot, where this sensitivity of roots could be attributed to the direct contact between roots and the allelochemicals or due to the permeability of root cells [42,43]. 

Significant variations between the EOs of the coastal and inland samples were observed for germination, root, and shoot growth (*p* = 0.0003, 0.0001, and 0.0001, respectively). The coastal sample of *H. curassavicum* EO showed more allelopathic effect on the *C. murale* compared to the inland samples, which could be ascribed to the variation in the quality and quantity of the chemical compounds of the EO (Table 2). The variation in the chemical composition of the EOs from coastal and inland samples could be ascribed to the variation in the habitat (Table 1), particularly the soil composition [3]. High content of salinity characterizes the soil of coastal areas (Table 1) due to the seepage of the seawater [42,44]. In *Thymus vulgaris*, the EO content was increased by 16.11% under the treatment by 100 mM NaCl [30].

The major compounds of the EO could participate either alone or in synergy in the allelopathic activity [5,31]. The EOs of the *H. curassavicum* showed the presence of hexahydrofarnesyl acetone, (-)-caryophyllene oxide, humulene oxide, farnesyl acetone, farnesyl acetone C, and nerolidol epoxy acetate as major compounds (Table 2). Although, hexahydrofarnesyl acetone is well known to possess antimicrobial [45,46], antioxidant [8,47,48], and insecticidal effects [38], its allelopathic activity is still not explored well [3]. Hexahydrofarnesyl acetone was identified as a major compound in the EOs from various plants, where hexahydrofarnesyl acetone represented 62.3% of the EO in *Sagittaria trifolia* [46], 56.30% in *Limonium bonduellei* [49], 38.20% in *Hildegardia barteri* [50], 37.50% in *Deinbollia pinnata*, 18.34% in *Equisetum arvense* [45], and 14.34% in *Otostegia persica* [8]. In this context, these studies referred to the biological activity of the EOs of these plants to the presence of hexahydrofarnesyl acetone in high concentration. In addition, (-)-caryophyllene oxide was reported to be responsible for the allelopathic activity of the EO from *Cullen plicata* [5,43].

#### 2.4.2. Allelopathic Effect of the MeOH Extract

**Figure 4:**
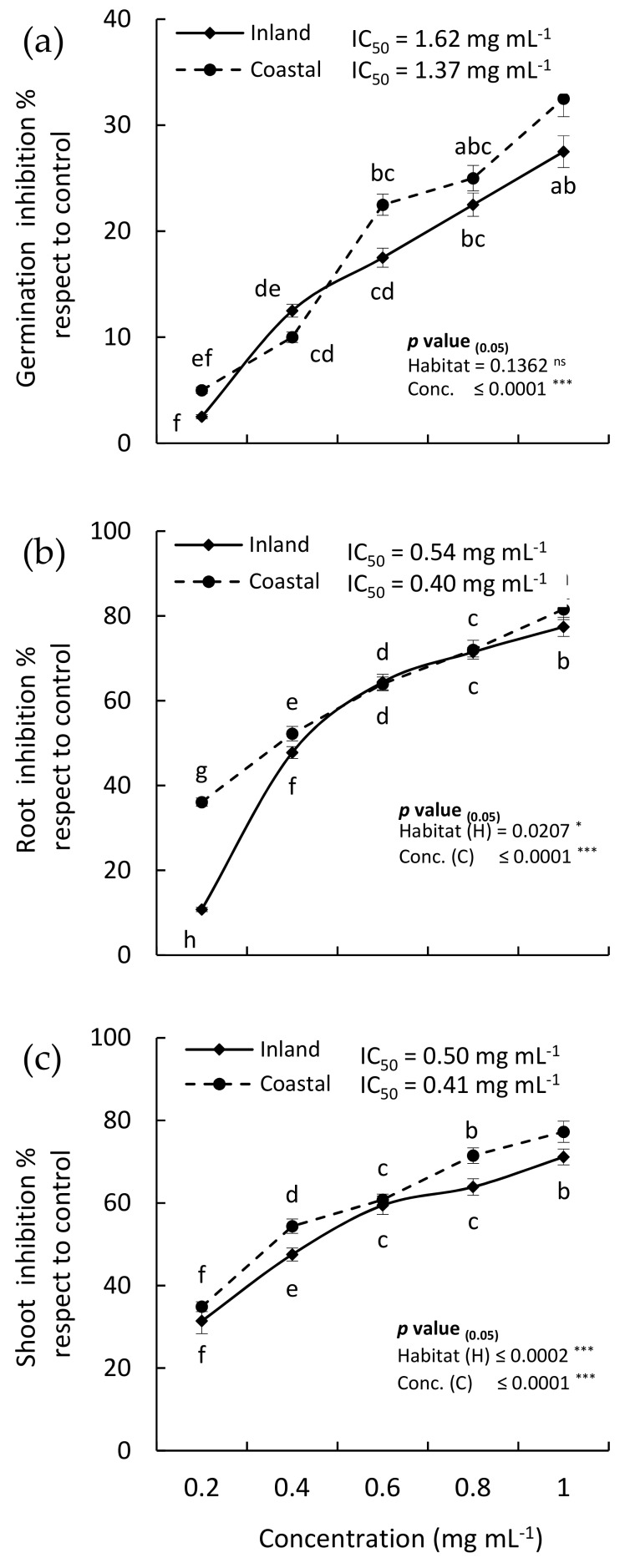
Allelopathic inhibitory effect of various concentrations of the MeOH extracts of the aerial parts of *Heliotropium curassavicum* collected from coastal and inland habitats on (**a**) the germination, (**b**) root, and (**c**) shoot growth of *Bidens pilosa*. Different letters within each measurement mean values of significant variation at *p* ≤ 0.05. IC_50_ is the concentration extract required for 50% inhibition.

The MeOH extracts from the coastal and inland revealed significant allelopathic activity against the weed, *C. murale* in a concentration-dependent manner (Figure 4). At the high concentration of the coastal sample extract (1 mg mL^−1^), the germination, root, and shoot growth of *C. murale* were reduced by 32.5%, 81.57%, and 77.24%, respectively, compared to control. While the inland samples revealed inhibition of *C. murale* by 27.5%, 77.42%, and 71.14%, respectively (Figure 4). The IC_50_ values of the coastal samples for germination, root, and shoot growth were 1.37, 0.40, and 0.41 mg mL^−1^, respectively. While the IC_50_ values of the inland sample were 1.62, 0.54, and 0.50 mg mL^−1^, respectively. Although no significant variation was observed between the coastal and inland samples (*p* = 0.1362), the growth of root and shoot revealed significant variation (*p* = 0.0207 and 0.0002, respectively). These variations could be attributed to the habitat effects. Additionally, the allelopathic activity of the coastal sample may be due to the high content of chlorogenic and gallic acids (Table 3). Phenolic acids are considered as the most common and effective allelochemicals in the ecosystem [51]. Phenolics diffuse into the environment and inhibit germination and growth when absorbed by plants [52]. The allelopathic effect of *Delonix regia* has been referred to a set of allelochemicals including chlorogenic and gallic acids [53]. In addition, chlorogenic acid was identified as allelochemical compounds in *Medicago sativa*, *Chenopodium album*, and *Xanthium strumarium* [54,55].

On the other hand, the allelopathic activity of the inland samples could be attributed to a mixture of bioactive compounds that were reported as allelochemicals such as vanillin, 4′,7-dihydroxyisoflavone, quercetin, caffeic, gallic, coumaric, and cinnamic acids (Table 3). El-Shora and Abd El-Gawad [56] reported the allelopathic activity of caffeic, gallic, coumaric, and cinnamic acids on the growth of *Cicer arietinum* and revealed the induction of various antioxidant enzymes (superoxide dismutase, catalase, and peroxidase) as well as the reactive oxygen species (ROS) and lipid peroxidation in the treated plants.

*Chenopodium murale* is reported as a nuisance weed that competes with various crops such as rice [57], barley [58], wheat [59], and chickpea [60]. It is worth mentioning here that the synthetic herbicides, 2,4-dichlorophenoxyacetic acid (2, 4-D), 2,4,5-trichlorophenoxyacetic acid (2, 4, 5-T), and 2-methyl-4-chlorophenoxyacetic acid (MCPA) were reported to inhibit the seed germination of *C. murale* by 97.06%, 61.76%, and 50.59%, respectively, at a concentration of 1000 ppm [61]. In this context, the present results revealed that the EO and the MeOH extract from *H. curassavicum* showed lower activity (2- to 3-fold) compared to synthetic herbicides. However, EO and MeOH extracts are still considered as a promising resource, and eco-friendly bioherbicide against weeds such as *C. murale*.

In general, the coastal sample of the *H. curassavicum* EO showed a greater allelopathic effect than the inland samples, and the MeOH extract was stronger in allelopathic activity than the EO. This may be attributed to the effect of habitats, particularly that the coastal habitat is more stressful than the inland habitat due to the effect of salinity, where the plants produce more bioactive compounds in order to tolerate the harsh conditions [62,63].

### 2.5. Antioxidant activity 

The *H. curassavicum* EOs of the coastal and inland samples showed a significant scavenging activity of both 2,2-diphenyl-1-picrylhydrazyl (DPPH) and 2,2’-azino-bis(3-ethylbenzothiazoline-6-sulphonic acid (ABTS) in a concentration-dependent manner (Table 4). The IC_50_ value of the EO from the coastal sample was 30.78 and 22.70 mg mL^−1^ for DPPH and ABTS, respectively, while the inland sample showed IC_50_ values of 40.40 and 29.92 mg mL^−1^. On the other hand, the MeOH extract of *H. curassavicum* revealed comparable antioxidant activity to the EO activity (Table 4), where the coastal and inland MeOH extracts attained IC_50_ values of 27.51 and 36.57 mg mL^−1^, respectively, of DPPH scavenging and 23.12 and 27.41 mg mL^−1^ of ABTS scavenging.

Based on the data of IC_50_ values, the ascorbic acid (standard antioxidant) revealed 3-fold of the antioxidant compared to the EO of the coastal sample and 4-fold regarding the inland sample. However, the ascorbic acid showed 3-fold of the antioxidant activity than the MeOH extracts of both coastal and inland samples (Table 4). The predominance of oxygenated sesquiterpenes (71.36% and 66.51%, for coastal and inland samples) could be the main factor for the considerable observed antioxidant activity of *H. curassavicum* EOs. Sesquiterpenoid compounds were reported as antioxidant compounds from various plants such as *Launaea* species [3], *Artemisia macrocephala* [7], and *Ferula caspica* [6]. The major compound hexahydrofarnesyl acetone was reported as a strong antioxidant compound from various plants such as *Otostegia persica* [8], *Launaea* species [3], *Impatiens* species [48], and *Stachys palustris* [47]. In addition, (-)-caryophyllene oxide was reported to be responsible for the antioxidant activity of the EO from *Cullen plicata* [42] and *Rhynchosia minima* [43]. 

In addition, the antioxidant activity of the MeOH extracts of coastal and inland samples could be corroborated to the high content of phenolic compounds and flavonoids (Table 3), especially with free aromatic hydroxyl groups. These bioactive compounds act as antioxidants that mitigate the effect oxidative stress and scavenge the ROS [56].

In general, the coastal samples expressed more antioxidant activity than the inland samples, as well as the MeOH extract was stronger than the EO. This difference in the activity between the coastal and inland could be corroborated to the effect of the habitat and environmental conditions [3,22].

Several documented studies confirmed the role of phenolic compounds as antioxidants such as phenolic acids and flavonoids. Hammad et al. [64] described that the combination of the hydrogen atom and electron transfer is the main antioxidant action pathway. In DPPH assay, the reactions with free OH in the B-ring of flavonoids or aromatic acids are the main discrimination between the phenolic compound and flavonoids [65]. By more analysis of the correlation of the antioxidant activity and the phenolic compositions, we can find that the inland MeOH extract is less active than the coastal one, although the inland sample is richer with phenolic compounds than the coastal one. According to the previously described mechanism of action of phenolic compounds as antioxidant agents, the results deduced that the concentration of the phenolic compounds and flavonoids, especially with free OH, in the inland MeOH extract is higher than the coastal one. The results exhibited that chlorogenic acid and rutin are the main components of the inland sample with significant concentration, while the non-free phenolic compounds are the main components of the coastal one. Thus, these results were in agreement with the fact of the direct relationship of the antioxidant potentiality and the numbers and concentrations of free OH groups [64,65]. 

## 3. Material and Methods

### 3.1. Plant Material

The aerial parts of *H. curassavicum*, as a composite sample, were collected during the flowering stage from two different habitats; the first location was sandy and saline habitat near Gamasa City, northern Mediterranean coast, Egypt (31°27′36.9″N 31°27′08″E), and this sample was named coastal sample. While the second location was fallow land habitat with clay soil near Mansoura University, Mansoura, Egypt (31°02′33.7″N 31°20′57.7″E), and this sample was named inland sample. The collected plant was identified according to Boulos [66] by the author, Associate Prof. Ahmed Abd-ElGawad. Voucher specimens (Mans.0021103005 and Mans.0021103006) were deposited in the herbarium of the Botany Department, Faculty of Science, Mansoura University, Egypt.

### 3.2. Soil Analyses

From each location (coastal and inland), rhizospheric soil samples from five patches of *H. curassavicum* were collected in plastic bags and brought to the laboratory. The collected soil was dried in room temperature at 25 ± 2°C, ground, sieved via 2 mm sieve, and packed until further analysis. Soil texture was determined by sieve method according to Piper [67], while organic carbon content was measured based on the modified Walkley–Black method [68]. Soil suspension (1:2.5) was prepared and pH was determined using the pH meter (Model: YK-2001PH, Lutron, Malaysia), while EC was measured by the conductivity meter (Model: CD-4306, Lutron, Malaysia). The total nitrogen of the soil was estimated using the micro-Kjeldahl method [69], while total dissolved phosphorus was measured spectrophotometrically according to APHA [70]. 

### 3.3. Extraction and Identification of EOs

The EOs of the fresh aerial parts (150 gm) of the two samples of *H. curassavicum* (coastal (0.026 mL) and inland (0.021 mL)) were extracted by hydro-distillation using a Clevenger-type apparatus for 3 h. The oily layer was separated using diethyl ether and dried with anhydrous sodium sulfate (0.5 g). This extraction was repeated two times and afforded two samples of EO for each plant sample. The extracted EOs of the two samples were stored in sealed air-tight glass vials at 4 °C until further analysis.

The EOs components of the extracted samples were analyzed separately and identified depending upon GC/MS analysis. The GC-MS analysis of the EO samples were carried out using a gas chromatography-mass spectrometry instrument at the Medicinal and Aromatic Plants Research Department, National Research Center, Dokki, Giza, Egypt. The instrument had the following specifications: TRACE GC Ultra Gas Chromatographs (THERMO Scientific™ Corporate, USA), lined with a Thermo Scientific ISQ™ EC single quadrupole mass spectrometer. The GC-MS system was equipped with a TR-5 MS column with a dimension of 30 m × 0.32 mm i.d., 0.25 μm film thickness. The analyses were achieved using helium as carrier gas at a flow rate of 1.0 mL/min with a split ratio of 1:10 using the following temperature program: 60 °C for 1 min; rising at 4.0 °C/min to 240 °C and held for 1 min. Both injector and detector were held at 210 °C. An aliquot of 1 μL of diluted samples in hexane (1:10, v/v) were always injected. Mass spectra were recorded by electron ionization (EI) at 70 eV, using a spectral range of *m/z* 40–450.

Chemical constituent’s identification of the EOs was deconvoluted using AMDIS software (www.amdis.net), retention indexes (relative to *n*-alkanes C_8_-C_22_), comparison of the mass spectrum with authentic standards (when available), and Wiley spectral library collection and NSIT library database.

### 3.4. Preparation of MeOH Extract and HPLC Analysis

About 100 g of air-dried powder aerial parts of the two samples of *H. curassavicum* (Coastal and Inland) were extracted in 70% hydro-methanol at room temperature (27 ± 2°C), filtered, and dried under vacuum to give dark black gum (1.6 and 1.85 g, respectively). 

The standard phenolic, gallic, cinnamic, chlorogenic, ferulic, coffeic, syringic, ellagic, coumaric acids, vanillin, caffeine, propyl gallate, and flavonoids, quercetin, rutin, catechin, pyrocatechol, naringenin, 4‘.7-dihydroxy isoflavone, were purchased from Sigma-Aldrich (Germany). Trifloroacetic acid and acetonitrile (HPLC gradient grades) were purchased from Sigma-Aldrich (Germany). The used di-distilled water in HPLC was obtained by Hamilton water distillation apparatus (Hamilton Laboratory Glass Ltd., Kent, England).

HPLC analysis was performed using an Agilent 1260 series. The separation was carried out using a C18 column (4.6 × 250 mm i.d., 5 μm). The mobile phase contained water (A) and 0.02% trifloroacetic acid in acetonitrile (B) with a flow rate of 1 mL min^−1^. The mobile phase was automated successively in a linear gradient as follows: 0 min (80% A), 0–5 min (80% A), 5–8 min (40% A), 8–12 min (50% A), 12–14 min (80% A), and 14–16 min (80% A). The multi-wavelength detector was monitored at 280 nm. The injection volume was 10 μL for each of the sample solutions. The column temperature was maintained at 35 °C.

### 3.5. Allelopathic Activity

The allelopathic activity of the EOs and the MeOH extract of *H. curassavicum* aerial parts was assessed against the weed, *C. murale*. The ripe seeds of this weed were collected from cultivated fields with wheat in Manzalla city, Dakahlia Governorate, Egypt (31°07′06.9′’ N, 31°51′53.3′’ E). Seeds were surface sterilized by 0.3% sodium hypochlorite for 3 min, and then washed with distilled and sterilized water, and dried over sterilized filter paper. To test the allelopathic activity of the EOs, concentrations of 0.2, 0.4, 0.6, 0.8, and 1.0 mg mL^−1^ were prepared using dimethyl sulfoxide (DMSO) (Sigma-Aldrich, Germany). On the other hand, similar concentrations (0.2, 0.4, 0.6, 0.8, and 1.0 mg mL^−1^) of the residue from the MeOH extract (its preparation is mentioned above) were prepared in DMSO. Subsequently, 20 *C. murale* seeds were arranged in Petri plates (Ø: 9 cm), lined with a Whatman No. 1 filter paper, and then 4 mL of each concentration was added. The experiment was designed with three replications, and a control treatment with either DMSO instead of the EO or MeOH extract. The plates sealed with a Parafilm^®^ tape (Sigma, USA), and incubated at 25 ± 2 °C in a growth chamber with a controlled light cycle of 16 h light and 8 h dark. After seven days of incubation, the germinated seeds were counted as well as the root and shoot length of all seedlings were measured. The inhibition of either germination or seedling length was calculated as follows:

(1) $$\mathrm{Inhibition}\;\left(\%\right)=100\times \frac{\left(\mathrm{No}/\mathrm{Length}\;\mathrm{of}\;\mathrm{control}-\mathrm{No}/\mathrm{Length}\;\mathrm{of}\;\mathrm{tretamnet}\right)}{\mathrm{No}/\mathrm{Length}\;\mathrm{of}\;\mathrm{control}}.$$

### 3.6. Antioxidant Activity

Either the EO or the MeOH extract from *H. curassavicum* aerial parts were examined for the antioxidant activity via DPPH and ABTS methods.

#### 3.6.1. DPPH Radical Scavenging Activity

The ability of EO and MeOH extracts to reduce the color of the DPPH radical (Sigma-Aldrich, Germany) were determined according to Miguel [71]. Various concentrations (5, 10, 20, 30, 40, and 50 mg mL^−1^) of the EOs and residues of the MeOH extract were prepared using methanol. This range of concentrations was determined based on a preliminary test using either higher concentration or lower concentration. To assess the antioxidant activity, a reaction mixture of equal volumes from the freshly prepared 0.3 mM DPPH and each concentration of the EOs or the MeOH extract, was prepared, mixed vigorously, and kept in dark for 15 min at 25 °C. Additionally, a parallel positive control, using ascorbic acid as a standard antioxidant, at concentrations of 1.0, 2.5, 5, 10, 15, and 20 mg mL^−1^, were prepared and treated similar to the treatments. After incubation, the absorbance was measured at 517 nm using a spectrophotometer (Milton Roy Spectronic 21D UV-Visible Spectrophotometer, USA). The amount of EO or MeOH extract required to reduce the color of DPPH by 50% (IC_50_) was calculated graphically.

#### 3.6.2. ABTS-Free Radical Scavenging Activity

In order to emphasize the antioxidant activity of the *H. curassavicum* EOs and MeOH extracts, the capability of scavenging ABTS radical (Sigma-Aldrich, Germany) was assessed following the method of Re et al. [72]. In brief, the radical was prepared by mixing 7 mM of ABTS (1/1, v/v) with 2.45 mM of potassium persulfate and kept in a dark condition at room temperature (25 ± 2 °C) for 16 h. ABTS radical was diluted by using methanol to reach the absorbance of 0.700 ± 0.02 at 734 nm. The reaction mixture of 0.2 mL of each concentration of the EO (5, 10, 15, 20, and 25 µL L^−1^) and 2 mL of the ABTS solution was prepared, mixed vigorously, left for 6 min at room temperature, and then the absorbance was measured at 734 nm. Ascorbic acid as natural antioxidant was used as a positive control. The percentage of scavenging and IC50 (the concentration of the EO which scavenge 50% of the ABTS radical) were calculated as previously mentioned in the DPPH assay.

### 3.7. Statistical Analysis

The allelopathy and antioxidant experiments were repeated three times (three replications). In each experiment, the measurements were measured in triplicate. The data were subjected to one-way analysis of variance, followed by Duncan’s test at probability level 0.05 using COSTAT software program (CoHort Software, Monterey, CA, USA). The soil variable data were subjected two-tailed *t*-test at the probability level of 0.05 using XLSTAT 2018 (Addinsoft, New York, NY, USA).

## 4. Conclusions

The GC-MS analysis of *H. curassavicum* EOs revealed the presence of 56 compounds (25 for the coastal sample and 52 from the inland one). The major compounds were hexahydrofarnesyl acetone, (-)-caryophyllene oxide, humulene oxide, farnesyl acetone, farnesyl acetone C, nerolidol epoxy acetate. On the other hand, HPLC of the phenolic profile of MeOH extracts of the two samples revealed the predominance of chlorogenic acid, rutin, and propyl gallate are majors in the coastal sample, while vanilin, quercetin, 4′,7-dihydroxyisoflavone are majors in the inland one. These variations in the EOs, phenolics, or flavonoids reflect the effect of the habitat as well as adaptability of the plant to different environmental conditions by shifting its chemical composition. The coastal sample of the *H. curassavicum* EO expressed more allelopathic and antioxidant activities than the inland samples, and the MeOH extract was stronger than the EO. These variations could be referred to habitat effects, especially as the coastal habitat is stressful than the inland habitat due to salinity, where the plants cope with harsh conditions via the enhancement of bioactive compound production. The bioactivity of the EO could be ascribed to the major compounds, particularly hexahydrofarnesyl acetone and (-)-caryophyllene oxide, while the bioactivity of the MeOH extract may be ascribed to chlorogenic acid, vanillin, quercetin, and propyl gallate. Although, the allelopathic activity of the EO and MeOH extracts was lower than the common synthetic herbicides, they are still a promising resource for an environmentally friendly bioherbicide for weed control, at least against *C. murale*.

## Figures and Tables

**Table 1:** Soil physical and chemical characteristics of the rhizospheric samples of *Heliotropium curassavicum* in coastal and inland habitats.

Parameter	Habitat		*p*-value
	Coastal	Inland	
Sand (%)	94.16 ± 2.05	67.39 ± 2.37	0.008 ^*^
Silt (%)	4.20 ± 1.79	19.56 ± 2.63	0.026 ^*^
Clay (%)	1.65 ± 0.68	13.06 ± 1.62	0.010 ^*^
pH	7.37 ± 0.49	7.17 ± 0.12	0.591
OC (g kg^−1^)	0.63 ± 0.09	1.30 ± 0.13	0.032 ^*^
EC (mS cm^−1^)	1.68 ± 0.21	0.57 ± 0.01	0.012 ^*^
TDP (g kg^−1^)	0.46 ± 0.10	2.11 ± 0.35	0.022 ^*^
TN (g kg^−1^)	0.33 ± 0.05	3.58 ± 1.04	0.033 ^*^

OC: organic carbon, EC: electrical conductivity, TDP: total dissolved phosphorus, TN: total nitrogen. ^*^ significant variation at the probability level of 0.05.

**Table 2:** Essential oil constituents of the coastal and inland samples of *Heliotropium curassavicum*.

No	Rt	KI_lit_^[a]^	KI_exp_^[b]^	Compound	Concentration % ^[c]^		Identification ^[d]^
					Coastal	Inland	
Monoterpene hydrocarbons							
1	4.45	936	936	*α*-Pinene	0.79 ± 0.03	0.4 ± 0.02	MS, KI
2	18.99	1375	1374	*β*-Elemene	--	0.32	MS, KI
Oxygenated Monoterpenes							
3	18.31	1173	1174	Menthol	0.86 ± 0.03	0.42 ± 0.02	MS, KI
4	25.42	1426	1425	trans-*α*-Ionone	--	2.05	MS, KI
5	25.57	1426	1426	*cis*-*α*-Ionone	--	2.21	MS, KI
6	40.99	1162	1164	*trans*-p-Menthan-7-ol	2.34 ± 0.05	1.81 ± 0.04	MS, KI
Sesquiterpene hydrocarbons							
7	20.38	1373	1373	*α*-Ylangene	--	0.27 ± 0.01	MS, KI
8	20.69	1376	1377	*α*-Copaene	--	0.51 ± 0.02	MS, KI
9	21.23	1351	1351	*α*-Cubebene	--	0.41 ± 0.01	MS, KI
10	21.83	1409	1410	*α*-Gurjunene	0.65 ± 0.03	1.09 ± 0.04	MS, KI
11	22.41	1442	1441	(+)-1(10)-Aristolene	--	2.92 ± 0.06	MS, KI
12	22.50	1417	1417	Isocaryophyllene	1.35 ± 0.06	--	MS, KI
13	22.53	1430	1431	*trans*-Caryophyllene	--	1.58 ± 0.04	MS, KI
14	24.20	1706	1705	Farnesane	1.51 ± 0.05	0.73 ± 0.03	MS, KI
15	24.85	1493	1493	*β*-Muurolene	--	1.67 ± 0.04	MS, KI
16	25.89	1499	1500	*α*-Muurolene	1.59 ± 0.03	0.78 ± 0.02	MS, KI
17	33.07	1460	1361	Dihydroaromadendrene	5.21 ± 0.06	2.71 ± 0.04	MS, KI
Oxygenated Sesquiterpenes							
18	20.99	1680	1682	6-epi-shyobunol	--	0.99 ± 0.02	MS, KI
19	21.53	--	1675	Isocaucalol	--	0.38 ± 0.01	MS
20	24.25	1675	1677	*Cis*-Z-*α*-Bisabolene epoxide	--	0.45 ± 0.01	MS, KI
21	24.95	1597	1578	Widdrol	--	0.43 ± 0.02	MS, KI
22	26.31	1632	1632	Nerolidyl acetate	--	0.48 ± 0.02	MS, KI
23	26.60	1494	1496	4-epi-cubedol	--	0.88 ± 0.03	MS, KI
24	27.32	1576	1577	Spathulenol	--	0.72 ± 0.01	MS, KI
25	29.20	1580	1580	Caryophyllene oxide	6.41 ± 0.05	7.86 ± 0.08	MS, KI
26	30.33	1605	1606	Humulene oxide	5.01 ± 0.04	4.02 ± 0.04	MS, KI
27	30.66	1584	1583	Isoaromadendrene epoxide	--	0.48 ± 0.02	MS, KI
28	30.83	1657	1658	Cyclolongifolene oxide, dehydro-	1.34 ± 0.03	0.99 ± 0.01	MS, KI
29	31.09	1648	1650	Aromadendrene oxide-(1)	--	0.71 ± 0.01	MS, KI
30	33.43	1683	1681	*α*-Bisabolol	--	1.42 ± 0.03	MS, KI
31	34.41	1563	1562	Hexahydrofarnesol	--	1.08 ± 0.03	MS, KI
32	38.72	1845	1845	Hexahydrofarnesyl acetone	50.39 ± 0.11	35.82 ± 0.09	MS, KI
33	39.00	--	1863	Nerolidol epoxy acetate	3.0 ± 0.07	1.64 ± 0.02	MS
34	41.27	1918	1919	Farnesyl acetone	--	7.17 ± 0.05	MS, KI
35	41.72	1921	1921	Farnesyl acetone C	5.21 ± 0.06	0.99 ± 0.02	MS, KI
Diterpene hydrocarbons							
36	25.06	1942	1943	Phytol	1.83 ± 0.04	1.05 ± 0.03	MS, KI
Oxygenated diterpenes							
37	26.92	1811	1812	Phytan	1.07 ± 0.04	0.72 ± 0.01	MS, KI
Non-oxygenated hydrocarbons							
38	16.28	1400	1400	*n*-Tetradecane	0.74 ± 0.02	--	MS, KI
39	16.32	1500	1498	*n*-pentadecane	--	0.32 ± 0.01	MS, KI
40	34.70	1555	1556	2,6,10-Trimethyltetradecane	1.02 ± 0.04	1.84 ± 0.05	MS, KI
41	40.28	1792	1792	Hexadecane, 2,6,11,15-tetramethyl (Crocetane)	2.31 ± 0.06	0.55 ± 0.02	MS, KI
42	40.48	1900	1902	*n*-Nonadecane	--	1.17 ± 0.03	MS, KI
43	43.74	2000	2001	*n*-Eicosane	--	0.80 ± 0.02	MS, KI
44	46.88	2100	2100	*n*-Heneicosane	3.31 ± 0.05	2.15 ± 0.06	MS, KI
45	47.07	2200	2200	*n*-Docosane	0.95 ± 0.02	--	MS, KI
47	52.86	2400	2401	*n*-Tetracosane	1.38 ± 0.02	0.91 ± 0.01	MS, KI
46	49.84	3500	3500	*n*-Pentatriacontane	--	0.61 ± 0.02	MS, KI
Oxygenated hydrocarbons							
48	31.53	1847	1851	2-Hexadecanol	1.07 ± 0.03	--	MS, KI
50	37.11	2760	2758	2-Hexyl-1-decanol	--	0.59 ± 0.02	MS, KI
51	37.47	2019	2019	11- Octadecanal	--	0.98 ± 0.02	MS, KI
52	40.59	2088	2088	Octadecanol	--	1.05 ± 0.03	MS, KI
53	48.07	2140	2143	9, 12-Octadecadienoic acid	4.43 ± 0.06	0.47 ± 0.01	MS, KI
Others							
54	17.19	1300	1302	Dihydroedulan II	0.66 ± 0.02	0.32 ± 0.01	MS, KI
55	32.07	--	1667	2-Tetra-butyl-4-isopropyl-5-methylphenol	--	0.55 ± 0.01	MS
Monoterpene hydrocarbons					0.79	0.72	
Oxygenated monoterpenes					3.2	6.49	
Sesquiterpene hydrocarbons					10.31	12.67	
Oxygenated sesquiterpenes					71.36	66.51	
Diterpene hydrocarbons					1.83	1.05	
Oxygenated diterpenes					1.07	0.72	
Non-oxygenated hydrocarbons					9.71	8.35	
Oxygenated hydrocarbons					1.07	2.62	
Others					0.66	0.87	
Total					100	100	

[**a**] KI: Kovats retention index from literature reviews, [**b**] KI: Experimental Kovates retention index, [**c**] values are mean ± standard deviation, and [**d**] the identification of essential oil components was established depending upon the mass spectral data of compounds (MS) and Kovats indices (RI) with those of Wiley spectral library collection and NIST library databases.

**Table 3:** Identified phenolic and flavonoid compounds of MeOH extract from *Heliotropium curassavicum* derived by HPLC.

No	Retention Time	Compound	Concentration (µg g^−1^)	
			Coastal Sample	Inland Sample
**Phenolics**				
1	3.114	Gallic Acid	69.66 ± 1.14	122.11 ± 1.21
2	3.441	Chlorogenic acid	1198.24 ± 2.42	190.23 ± 1.32
3	3.893	Caffeine	26.03 ± 1.12	51.45 ± 1.22
4	4.798	Caffeic Acid	--	452.87 ± 2.67
5	5.195	Syringic acid	16.97 ± 0.92	96.91 ± 1.18
6	5.496	Rutin	728.44 ± 2.92	199.63 ± 1.56
7	6.655	Ellagic acid	22.84	--
8	7.453	Coumaric Acid	--	95.36 ± 1.02
9	8.05	Vanillin	100.63 ± 2.06	1284.61 ± 3.08
10	11.118	Cinnamic Acid	--	163.66 ± 1.13
**Flavonoids**				
11	3.678	Catechin	36.02 ± 0.82	270.76 ± 1.82
12	9.329	Naringenin	--	120.56 ± 1.41
13	10.221	Propyl gallate	133.09 ± 1.39	233.67 ± 2.17
14	10.442	4′,7-Dihydroxyisoflavone	42.19 ± 0.77	721.52 ± 3.16
15	10.628	Quercetin	--	603.63 ± 2.97

**Table 4:** Scavenging activity percentage of 2,2-Diphenyl-1-picrylhydrazyl (DPPH) and 2,2’-azino-bis(3-ethylbenzothiazoline-6-sulphonic acid (ABTS) as well as the IC_50_ values by *Heliotropium curassavicum* essential oil and the MeOH extract as well as ascorbic acid as standard.

Treatment	Conc. (mg mL^−1^)	DPPH		ABTS	
		Coastal	Inland	Coastal	Inland
Essential oil extract	50	72.83 ± 1.63^*A^	63.44 ± 3.10^B^	76.11 ± 1.38^A^	69.30 ± 1.68^B^
	40	62.57 ± 2.48^B^	47.69 ± 2.87^C^	68.49 ± 0.99^B^	64.92 ± 1.45^C^
	30	49.12 ± 1.79^C^	35.89 ± 1.86^D^	61.62 ± 1.53^D^	49.41 ± 0.46^E^
	20	33.92 ± 2.02^D^	26.12 ± 3.42^E^	53.14 ± 1.76^F^	38.49 ± 1.22^G^
	10	26.78 ± 1.40^E^	20.19 ± 0.47^F^	37.62 ± 1.53^G^	31.30 ± 1.15^H^
	5	18.99 ± 1.55^F^	9.00 ± 1.86^G^	23.68 ± 0.92^I^	19.78 ± 0.46^J^
IC_50_ (mg mL^−1^)		30.78	40.40	22.70	29.92
*F* value		173.33		444.17	
MeOH extract	50	73.20 ± 1.54^A^	65.27 ± 1.03^B^	77.96 ± 1.31^A^	70.03 ± 1.46^B^
	40	61.17 ± 1.69^C^	52.46 ± 1.69^D^	66.37 ± 1.24^C^	62.62 ± 1.02^D^
	30	53.81 ± 1.98^D^	43.39 ± 1.32^E^	59.11 ± 1.17^E^	57.11 ± 1.24^E^
	20	45.20 ± 1.69^E^	32.97 ± 1.25^F^	48.51 ± 1.60^F^	45.26 ± 0.80^G^
	10	34.11 ± 1.25^F^	21.31 ± 1.03^H^	37.85 ± 0.80^H^	33.42 ± 1.09^I^
	5	25.51 ± 1.10^G^	9.59 ± 0.59^I^	27.50 ± 0.73^J^	21.83 ± 0.58^K^
IC_50_ (mg mL^−1^)		27.00	36.57	23.12	27.41
*F* value		372.68		499.06	
Ascorbic acid	20	66.63 ± 1.02^A^		77.90 ± 1.42^A^	
	15	57.89 ± 0.98^B^		60.97 ± 1.12^B^	
	10	46.85 ± 0.65^C^		49.52 ± 0.86^C^	
	5	39.86 ± 0.45^D^		37.01 ± 0.86^D^	
	2.5	8.99 ± 0.05^E^		13.09 ± 0.80^E^	
	1	2.00 ± 0.03^F^		4.53 ± 0.28^F^	
IC_50_ (mg mL^−1^)		12.96		11.52	
*F* value		318.15		299.25	

* values are means of triplicate ± standard deviation, IC_50_ is the concentration of the sample that was required to reduce the DPPH absorbance by 50%. Different superscript letters within each column mean values of significant variation at *p* < 0.05.

## References

[1] Hussain K., Shahazad A., Zia-ul-Hussnain S. (2008). An ethnobotanical survey of important wild medicinal plants of Hattar district Haripur, Pakistan. Ethnobot. Leafl..

[2] David B., Wolfender J.-L., Dias D.A. (2015). The pharmaceutical industry and natural products: Historical status and new trends. Phytochem. Rev..

[3] Elshamy A., Abd-ElGawad A.M., El-Amier Y.A., El Gendy A., Al-Rowaily S. (2019). Interspecific variation, antioxidant and allelopathic activity of the essential oil from three *Launaea* species growing naturally in heterogeneous habitats in Egypt. Flavour Fragr. J..

[4] Sharifi-Rad J., Sureda A., Tenore G.C., Daglia M., Sharifi-Rad M., Valussi M., Tundis R., Sharifi-Rad M., Loizzo M.R., Ademiluyi A.O. (2017). Biological activities of essential oils: From plant chemoecology to traditional healing systems. Molecules.

[5] Abd El-Gawad A.M. (2016). Chemical constituents, antioxidant and potential allelopathic effect of the essential oil from the aerial parts of *Cullen plicata*. Ind. Crops Prod..

[6] Kahraman C., Topcu G., Bedir E., Tatli I.I., Ekizoglu M., Akdemir Z.S. (2019). Phytochemical screening and evaluation of the antimicrobial and antioxidant activities of *Ferula caspica* M. Bieb. extracts. Saudi Pharm. J..

[7] Shoaib M., Shah I., Ali N., Adhikari A., Tahir M.N., Shah S.W.A., Ishtiaq S., Khan J., Khan S., Umer M.N. (2017). Sesquiterpene lactone! A promising antioxidant, anticancer and moderate antinociceptive agent from *Artemisia macrocephala* jacquem. BMC Complement. Altern. Med..

[8] Tofighi Z., Alipour F., Yassa N., Hadjiakhoondi A., Hadavinia H., Goodarzy S., Golestani R. (2009). Chemical composition and antioxidant activity of *Otostegia persica* essential oil from Iran. Int. J. Essent. Oil Ther..

[9] Sakr A.A., Ghaly M.F., Abdel-Haliem M.E.-S.F. (2012). The efficacy of specific essential oils on yeasts isolated from the royal tomb paintings at Tanis, Egypt. Int. J. Conserv. Sci..

[10] Rotolo V., De Caro M., Giordano A., Palla F. (2018). Solunto archaeological park in Sicily: Life under mosaic tesserae. Flora Medit.

[11] Walentowska J., Foksowicz-Flaczyk J. (2013). Thyme essential oil for antimicrobial protection of natural textiles. Int. Biodeter. Biodegr..

[12] Abd El-Gawad A.M., El Gendy A.G., Elshamy A.I., Omer E.A. (2016). Chemical composition of the essential oil of *Trianthema portulacastrum* L. Aerial parts and potential antimicrobial and phytotoxic activities of its extract. J. Essent. Oil Bear. Plants.

[13] Bruin J., Sabelis M.W., Dicke M. (1995). Do plants tap SOS signals from their infested neighbours?. Trends Ecol. Evol..

[14] Miresmailli S., Isman M.B. (2014). Botanical insecticides inspired by plant—Herbivore chemical interactions. Trends Plant Sci..

[15] Cseke L.J., Kaufman P.B., Kirakosyan A. (2007). The biology of essential oils in the pollination of flowers. Nat. Prod. Commun..

[16] Barney J.N., Tekiela D.R., Dollete E.S., Tomasek B.J. (2013). What is the “real” impact of invasive plant species?. Front. Ecol. Environ..

[17] Zheng Y.L., Feng Y.L., Zhang L.K., Callaway R.M., Valiente-Banuet A., Luo D.Q., Liao Z.Y., Lei Y.B., Barclay G.F., Silva-Pereyra C. (2015). Integrating novel chemical weapons and evolutionarily increased competitive ability in success of a tropical invader. New Phytol..

[18] Einhellig F.A. (1995). Allelopathy: Current Status and Future Goals.

[19] Al-Shehbaz I.A. (1991). The genera of Boraginaceae in the southeastern United States. J. Arnold. Arbor..

[20] Hegazy A., Mussa S., Farrag H. (2008). Invasive plant communities in the Nile Delta coast. Glob. J. Environ. Res..

[21] Hegazy A.K. (1994). Trade-off between sexual and vegetative reproduction of the weedy *Heliotropium curassavicum*. J. Arid Environ..

[22] Abd-ElGawad A.M., Elshamy A.I., El-Amier Y.A., El Gendy A.E.-N.G., Al-Barati S.A., Dar B.A., Al-Rowaily S.L., Assaeed A.M. (2019). Chemical composition variations, allelopathic, and antioxidant activities of *Symphyotrichum squamatum* (Spreng.) Nesom essential oils growing in heterogeneous habitats. Arab. J. Chem..

[23] Souza J.S.N., Machado L.L., Pessoa O.D., Braz-Filho R., Overk C.R., Yao P., Cordell G.A., Lemos T.L. (2005). Pyrrolizidine alkaloids from *Heliotropium indicum*. J. Braz. Chem. Soc..

[24] Yeo D., Attioua B., Lehalle C., Kossi M., N’guessan J.D., Djaman A.J., Lobstein A., Frossard N. (2011). Isolation of wound healing compounds from *Heliotropium indicum*. J. Appl. Pharm. Sci..

[25] Singh B., Sahu P.M., Sharma R.A. (2017). Flavonoids from *Heliotropium subulatum* exudate and their evaluation for antioxidant, antineoplastic and cytotoxic activities II. Cytotechnology.

[26] Singh B., Sahu P., Sharma R. (2018). In vitro and in vivo evaluation of flavonoids from *Heliotropium ellipticum* exudate for antioxidant, antineoplastic and cytotoxic activities ii. Indian J. Pharm. Sci..

[27] Jain S., Singh B., Jain R. (2001). Antimicrobial activity of triterpenoids from *Heliotropium ellipticum*. Fitoterapia.

[28] Modak B., Rojas M., Torres R. (2009). Chemical analysis of the resinous exudate isolated from *Heliotropium taltalense* and evaluation of the antioxidant activity of the phenolics components and the resin in homogeneous and heterogeneous systems. Molecules.

[29] Khan H., Khan M.A., Gul F., Hussain S., Ashraf N. (2015). Anti-inflammatory activity of *Heliotropium strigosum* in animal models. Toxicol. Ind. Health.

[30] Cordovilla M.P., Bueno M., Aparicio C., Urrestarazu M. (2014). Effects of salinity and the interaction between *Thymus vulgaris* and Lavandula angustifolia on growth, ethylene production and essential oil contents. J. Plant Nutr..

[31] Abd El-Gawad A.M., Elshamy A.I., El Gendy A.E.-N., Gaara A., Assaeed A.M. (2019). Volatiles profiling, allelopathic activity, and antioxidant potentiality of *Xanthium strumarium* leaves essential oil from Egypt: Evidence from chemometrics analysis. Molecules.

[32] Ogunbinu A.O., Flamini G., Cioni P.L., Adebayo M.A., Ogunwande I.A. (2009). Constituents of *Cajanus cajan* (L.) Millsp., *Moringa oleifera* Lam., *Heliotropium indicum* L. and *Bidens pilosa* L. from Nigeria. Nat. Prod. Commun..

[33] Abd-ElGawad A.M., Elshamy A., El Gendy A.E.-N., Al-Rowaily S.L., Assaeed A.M. (2019). Preponderance of oxygenated sesquiterpenes and diterpenes in the volatile oil constituents of *Lactuca serriola* L. revealed antioxidant and allelopathic activity. Chem Biodivers..

[34] Machan T., Korth J., Liawruangrath B., Liawruangrath S., Pyne S.G. (2006). Composition and antituberculosis activity of the volatile oil of *Heliotropium indicum* Linn. growing in Phitsanulok, Thailand. Flavour Fragr. J..

[35] Saeedi M., Morteza-Semnani K. (2009). Chemical composition and antimicrobial activity of the essential oil of *Heliotropium europaeum*. Chem. Nat. Compd..

[36] Abd El-Gawad A.M., El-Amier Y.A. (2015). Allelopathy and potential impact of invasive *Acacia saligna* (Labill.) Wendl. on plant diversity in the Nile Delta coast of Egypt. Int. J. Environ. Res..

[37] Nour V., Trandafir I., Cosmulescu S. (2012). HPLC determination of phenolic acids, flavonoids and juglone in walnut leaves. J. Chromatogr. Sci..

[38] Sotubo S.E., Lawal O.A., Osunsami A.A., Ogunwande I.A. (2016). Constituents and insecticidal activity of *Deinbollia pinnata* essential oil. Nat. Prod. Commun..

[39] Ghori M.K., Ghaffari M.A., Hussain S.N., Manzoor M., Aziz M., Sarwer W. (2016). Ethnopharmacological, phytochemical and pharmacognostic potential of genus *Heliotropium* L.. Turk. J. Pharm. Sci..

[40] Bistgani Z.E., Hashemi M., DaCosta M., Craker L., Maggi F., Morshedloo M.R. (2019). Effect of salinity stress on the physiological characteristics, phenolic compounds and antioxidant activity of *Thymus vulgaris* L. and *Thymus daenensis* Celak. Ind. Crops Prod..

[41] Salem N., Msaada K., Dhifi W., Limam F., Marzouk B. (2014). Effect of salinity on plant growth and biological activities of *Carthamus tinctorius* L. extracts at two flowering stages. Acta Physiol. Plant..

[42] Abd El-Gawad A.M. (2014). Ecology and allelopathic control of *Brassica tournefortii* in reclaimed areas of the Nile Delta, Egypt. Turk. J. Bot..

[43] Abd El-Gawad A.M., El-Amier Y.A., Bonanomi G. (2018). Allelopathic activity and chemical composition of *Rhynchosia minima* (L.) DC. essential oil from Egypt. Chem. Biodivers..

[44] Abd El-Gawad A.M., Shehata H.S. (2014). Ecology and development of *Mesembryanthemum crystallinum* L. in the Deltaic Mediterranean coast of Egypt. Egypt. J. Basic Appl. Sci..

[45] Radulović N., Stojanović G., Palić R. (2006). Composition and antimicrobial activity of *Equisetum arvense* L. essential oil. Phytother. Res..

[46] Xiangwei Z., Xiaodong W., Peng N., Yang Z., JiaKuan C. (2006). Chemical composition and antimicrobial activity of the essential oil of *Sagittaria trifolia*. Chem. Nat. Compd..

[47] Venditti A., Frezza C., Bianco A., Serafini M., Cianfaglione K., Nagy D.U., Iannarelli R., Caprioli G., Maggi F. (2017). Polar constituents, essential oil and antioxidant activity of marsh woundwort (*Stachys palustris* L.). Chem. Biodivers..

[48] Szewczyk K., Kalemba D., Komsta Ł., Nowak R. (2016). Comparison of the essential oil composition of selected *Impatiens* species and its antioxidant activities. Molecules.

[49] Benmeddour T., Laouer H., Flamini G., Akkal S. (2018). Chemical Composition of essential oil of *Limonium bonduellei*. Chem. Nat. Compd..

[50] Balogun O.S., Ajayi O.S., Adeleke A.J. (2017). Hexahydrofarnesyl acetone-rich extractives from *Hildegardia barteri*. J. Herbs Spices Med. Plants.

[51] Li Z.-H., Wang Q., Ruan X., Pan C.-D., Jiang D.-A. (2010). Phenolics and plant allelopathy. Molecules.

[52] Inderjit (1996). Plant phenolics in allelopathy. Bot. Rev..

[53] Chou C.-H., Leu L.-L. (1992). Allelopathic substances and interactions of *Delonix regia* (Boj) Raf. J. Chem. Ecol..

[54] Chon S.U., Kim J.D. (2002). Biological activity and quantification of suspected allelochemicals from alfalfa plant parts. J. Agron. Crop Sci..

[55] Mallik M., Puchala R., Grosz F. (1994). A growth inhibitory factor from lambsquarters (*Chenopodium album*). J. Chem. Ecol..

[56] El-Shora H.M., Abd El-Gawad A.M. (2014). Evaluation of allelopathic potential of root extract of *Rumex dentatus* and allelochemicals on *Cicer arietinum*. J. Stress Physiol. Biochem..

[57] Alam S., Shaikh A. (2007). Influence of leaf extract of nettle leaf goosefoot (*Chenopodium murale* L.) and NaCl salinity on germination and seedling growth of rice (*Oryza sativa* L.). Pak. J. Bot..

[58] Al-Johani N.S., Aytah A.A., Boutraa T. (2012). Allelopathic impact of two weeds, *Chenopodium murale* and *Malva parviflora* on growth and photosynthesis of barley (*Hordeum vulgare* L.). Pak. J. Bot..

[59] Majeed A., Chaudhry Z., Muhammad Z. (2012). Allelopathic assessment of fresh aqueous extracts of *Chenopodium album* L. for growth and yield of wheat (*Triticum aestivum* L.). Pak. J. Bot..

[60] Batish D., Lavanya K., Pal Singh H., Kohli R. (2007). Root-mediated allelopathic interference of nettle-leaved goosefoot (*Chenopodium murale*) on wheat (*Triticum aestivum*). J. Agron. Crop Sci..

[61] Agarwal S.K. (2009). Pesticide Pollution.

[62] Alam M.A., Juraimi A., Rafii M., Hamid A., Aslani F., Alam M. (2015). Effects of salinity and salinity-induced augmented bioactive compounds in purslane (*Portulaca oleracea* L.) for possible economical use. Food Chem..

[63] Vafadar Shoshtari Z., Rahimmalek M., Sabzalian M.R., Hosseini H. (2017). Essential oil and bioactive compounds variation in myrtle (*Myrtus communis* L.) as affected by seasonal variation and salt stress. Chem. Biodivers..

[64] Hammad H.M., Albu C., Matar S.A., Litescu S.-C., Al Jaber H.I., Abualraghib A.S., Afifi F.U. (2013). Biological activities of the hydro-alchoholic and aqueous extracts of *Achillea biebersteinii* Afan.(Asteraceae) grown in Jordan. Afr. J. Pharm. Pharm..

[65] El-Kashak W.A., Elshamy A.I., Mohamed T.A., El Gendy A.E.-N.G., Saleh I.A., Umeyama A. (2017). Rumpictuside A: Unusual 9, 10-anthraquinone glucoside from *Rumex pictus* Forssk. Carbohydr. Res..

[66] Boulos L. (1999). Flora of Egypt: Azollaceae-Oxalidaceae.

[67] Piper C.S. (1947). Soil and Plant Analysis.

[68] Walkley A., Black I.A. (1934). An examination of the Degtjareff method for determining soil organic matter, and a proposed modification of the chromic acid titration method. Soil Sci..

[69] Jackson M.L. (1973). Soil Chemical Analysis.

[70] APHA (1998). Standard Methods for the Examination of Water and Waste Water.

[71] Miguel M.G. (2010). Antioxidant activity of medicinal and aromatic plants. Flavour Fragr. J..

[72] Re R., Pellegrini N., Proteggente A., Pannala A., Yang M., Rice-Evans C. (1999). Antioxidant activity applying an improved ABTS radical cation decolorization assay. Free Radic. Biol. Med..

